# Supplementary material for: Dietary Conjugated Linoleic Acid Reduces Body Weight and Fat in Snord116m+/p− and Snord116m−/p− Mouse Models of Prader–Willi Syndrome
Source: Nutrients. 2022 Feb 18;14(4):860. doi: 10.3390/nu14040860 (PMC8880678; doi:10.3390/nu14040860)
Supplement: Supplementary file 1 [file nutrients-14-00860-s001.zip › Statistical Data Tables.pdf]

Statistical Data Tables

From JMP Statisical Software, Version Pro15
Red font = significant
Orange font = *P* less than 0.0001

Body weight by week

Source	Nparm	DF	Sum of Squares	F Ratio	Prob > F
Genotype	2	2	2415.0555	234.1928	<.0001*
Treatment	1	1	926.1966	179.6303	<.0001*
Week	12	12	541.3119	8.7487	<.0001*
Genotype*Treatment	2	2	46.2957	4.4894	0.0117*
Genotype*Week	24	24	48.7914	0.3943	0.9961
Treatment*Week	12	12	150.5177	2.4327	0.0044*
Genotype*Treatment*Week	24	24	58.8984	0.4760	0.9847

Body Weight-week 12

Source	Nparm	DF	Sum of Squares	F Ratio	Prob > F
Genotype	2	2	241.28281	17.3889	<.0001*
Treatment	1	1	162.47934	23.4193	<.0001*
Genotype*Treatment	2	2	12.09191	0.8714	0.4259

Fat Mass by week

Source	Nparm	DF	Sum of Squares	F Ratio	Prob > F
Genotype	2	2	153.72562	109.7742	<.0001*
Treatment	1	1	433.45607	619.0547	<.0001*
Week	12	12	18.02313	2.1450	0.0132*
Genotype*Treatment	2	2	45.68598	32.6240	<.0001*
Genotype*Week	24	24	10.97833	0.6533	0.8962
Treatment*Week	12	12	105.09223	12.5076	<.0001*
Genotype*Treatment*Week	24	24	19.49379	1.1600	0.2733

Fat Mass -week 12

Source	Nparm	DF	Sum of Squares	F Ratio	Prob > F
Genotype	2	2	17.470671	9.6695	0.0004*
Treatment	1	1	83.374886	92.2914	<.0001*
Genotype*Treatment	2	2	9.416571	5.2118	0.0096*

Lean Body Mass by week

Source	Nparm	DF	Sum of Squares	F Ratio	Prob > F
Genotype	2	2	375.88168	52.9455	<.0001*
Treatment	1	1	0.01110	0.0031	0.9554
Week	12	12	481.39952	11.3014	<.0001*

Source	Nparm	DF	Sum of Squares	F Ratio	Prob > F
Genotype*Treatment	2	2	34.13439	4.8081	0.0085*

Lean Body Mass-week 12

Source	Nparm	DF	Sum of Squares	F Ratio	Prob > F
Genotype	2	2	101.94217	15.2837	<.0001*
Treatment	1	1	2.75841	0.8271	0.3684
Genotype*Treatment	2	2	1.30931	0.1963	0.8225

Temperature-by week

Source	Nparm	DF	Sum of Squares	F Ratio	Prob > F
Genotype	2	2	1.108770	1.2263	0.2942
Treatment	1	1	1.180395	2.6110	0.1067
Week	12	12	10.030372	1.8489	0.0382*
Genotype*Treatment	2	2	0.010462	0.0116	0.9885
Genotype*Week	24	24	6.518275	0.6007	0.9341
Treatment*Week	12	12	4.271536	0.7874	0.6638
Genotype*Treatment*Week	24	24	7.420291	0.6839	0.8693

Temperature-week 12

Source	Nparm	DF	Sum of Squares	F Ratio	Prob > F
Genotype	2	2	0.7213672	0.9420	0.3981
Treatment	1	1	0.0514747	0.1344	0.7158
Genotype*Treatment	2	2	1.5644544	2.0429	0.1426

Temperature-whole study

Source	Nparm	DF	Sum of Squares	F Ratio	Prob > F
Genotype	2	2	1.894437	2.2221	0.1094
Treatment	1	1	2.026500	4.7541	0.0297*
Week	12	12	10.392803	2.0318	0.0200*
Genotype*Treatment	2	2	0.283136	0.3321	0.7176
Genotype*Week	24	24	8.144273	0.7961	0.7435
Treatment*Week	12	12	3.555686	0.6951	0.7568
Genotype*Treatment*Week	24	24	6.938580	0.6782	0.8745

Food intake-by week

Source	Nparm	DF	Sum of Squares	F Ratio	Prob > F
Genotype	2	2	972.0919	41.9005	<.0001*
Treatment	1	1	67.7568	5.8411	0.0160*
Week	11	11	1271.8840	9.9678	<.0001*

Source	Nparm	DF	Sum of Squares	F Ratio	Prob > F
Genotype*Treatment	2	2	21.9269	0.9451	0.3893
Genotype*Week	22	22	123.1850	0.4827	0.9785
Treatment*Week	11	11	63.8593	0.5005	0.9031
Genotype*Treatment*Week	22	22	150.2563	0.5888	0.9319

Food intake/body weight, by week

Source	Nparm	DF	Sum of Squares	F Ratio	Prob > F
Genotype	2	2	0.348	7.58	<.00006*
Treatment	1	1	1.236	53.768	<.0001*
Week	11	11	4.000	15.810	<.0001*
Genotype*Treatment	2	2	0.058	1.275	0.2802
Genotype*Week	22	22	0.205	0.406	0.9931
Treatment*Week	11	11	0.1433	0.5664	0.8563
Genotype*Treatment*Week	22	22	0.3670	0.725	0.815

Food intake-week 12

Source	Nparm	DF	Sum of Squares	F Ratio	Prob > F
Genotype	2	2	73.062538	2.4121	0.1029
Treatment	1	1	0.015142	0.0010	0.9749
Genotype*Treatment	2	2	12.499807	0.4127	0.6647

Fasting Glucose-Pre

Source	Nparm	DF	Sum of Squares	F Ratio	Prob > F
Genotype	2	2	4962.6770	4.2635	0.0202*

Fasting Glucose-Post

Source	Nparm	DF	Sum of Squares	F Ratio	Prob > F
Genotype	2	2	2329.489	0.7440	0.4815
Treatment	1	1	11718.166	7.4856	0.0092*
Genotype*Treatment	2	2	2002.489	0.6396	0.5327

Area under the Curve (Glucose tolerance)-Pre

Source	Nparm	DF	Sum of Squares	F Ratio	Prob > F
GENOTYPE	2	2	203267705	4.1907	0.0217*

Area under the Curve (Glucose tolerance)-Post

Source	Nparm	DF	Sum of Squares	F Ratio	Prob > F
GENOTYPE	2	2	76328543	0.5721	0.5689
TREATMENT	1	1	191679715	2.8731	0.0978
GENOTYPE*TREATMENT	2	2	203031326	1.5216	0.2307

Metabolism RER post (only)

Source	Nparm	DF	Sum of Squares	F Ratio	Prob > F
Genotype	2	2	0.00697954	2.0608	0.1469
Treatment	1	1	0.00003781	0.0223	0.8823
Genotype*Treatment	2	2	0.00016326	0.0482	0.9530

Metabolism EE post (only)

Source	Nparm	DF	Sum of Squares	F Ratio	Prob > F
Genotype	2	2	683.29358	0.9195	0.4109
Treatment	1	1	813.65946	2.1898	0.1505
Genotype*Treatment	2	2	434.79191	0.5851	0.5640

Running Wheel-Pre

Source	Nparm	DF	Sum of Squares	F Ratio	Prob > F
Genotype	2	2	5853024.7	3.1810	0.0510

Running Wheels-Post

Source	Nparm	DF	Sum of Squares	F Ratio	Prob > F
Genotype	2	2	40847854	9.3478	0.0005*
Treatment	1	1	2062316	0.9439	0.3370
Genotype*Treatment	2	2	17828236	4.0799	0.0242*

24-hr Home Cage Activity-post (only)

Source	Nparm	DF	Sum of Squares	F Ratio	Prob > F
Genotype	2	2	32341.628	2.2751	0.1221
Treatment	1	1	8607.440	1.2110	0.2808
Genotype*Treatment	2	2	12419.832	0.8737	0.4289

Rotorod-post (only)

Source	Nparm	DF	Sum of Squares	F Ratio	Prob > F
Genotype	2	2	10255.044	2.7049	0.0788
Treatment	1	1	5714.448	3.0146	0.0900
Genotype*Treatment	2	2	3718.096	0.9807	0.3837

Elevated Plus-open arms-Pre

Source	Nparm	DF	Sum of Squares	F Ratio	Prob > F
Genotype	2	2	3355.1057	0.7831	0.4635
Treatment	1	1	3042.2047	1.4202	0.2401
Genotype*Treatment	2	2	3979.5834	0.9289	0.4030

Elevated Plus-open arms-Post

Source	Nparm	DF	Sum of Squares	F Ratio	Prob > F
Genotype	2	2	13845.656	2.8814	0.0675

Source	Nparm	DF	Sum of Squares	F Ratio	Prob > F
Treatment	1	1	2.274	0.0009	0.9756
Genotype*Treatment	2	2	2594.691	0.5400	0.5869

Statistical data tables from GraphPad Prism 9.2.0
Red font = significant
Orange font = *P* less than 0.0001

Torque normalized to body mass (end of study)

Source	DF	Sum of Squares	F Ratio	Prob > F
Group	5	0.2844	16.42	<0.0001*
Frequency	10	13.18	380.6	<0.0001*
Interaction	50	0.07779	0.4491	0.9996

Torque normalized to lean body mass (end of study)

Source	DF	Sum of Squares	F Ratio	Prob > F
Group	5	0.1823	7.063	<0.0001*
Frequency	10	21.45	415.7	<0.0001*
Interaction	50	0.1424	0.5520	0.9945

Fatigue as percent of initial peak contractile torque (end of study)

Source	DF	Sum of Squares	F Ratio	Prob > F
Group	5	15948	30.46	<0.0001*
Contraction #	29	364825	120.1	<0.0001*
Interaction	145	3708	0.2442	>0.9999
