# Supplementary material for: Dietary Conjugated Linoleic Acid Reduces Body Weight and Fat in Snord116m+/p− and Snord116m−/p− Mouse Models of Prader–Willi Syndrome
Source: Nutrients. 2022 Feb 18;14(4):860. doi: 10.3390/nu14040860 (PMC8880678; doi:10.3390/nu14040860)

Supplemental Figures

Figure S1: *Whole study effects on weight, fat, lean mass, temperature, and food intake.* Post hoc analysis findings for (A) body weight -effect of genotype (both treatments), (B) body weight-effect of treatment (all genotypes), (C) body fat -effect of treatment (all genotypes), (D) lean mass-effect of genotype (all treatments), (E) temperature-effect of treatment (all genotypes), (F) food intake-effect of genotype (both treatments). (G) food intake/gram body weight-effect of genotype, and (H) food intake/gram body weight-effect of treatment. All data are presented as mean +/- standard error of the mean. N=8 WT control, 8 WT CLA, 8 PWS control, 7 PWS CLA, 7 PWS-KO control, 8 PWS-KO CLA. Letters indicate significant differences within a single figure for effects of genotype. For effect of treatment, ***P<0.001, *P<0.05. Individual statistics for each measure are provided in text, and as data tables in supplementary data files. ~~ used to indicate y-axis that are not at 0.

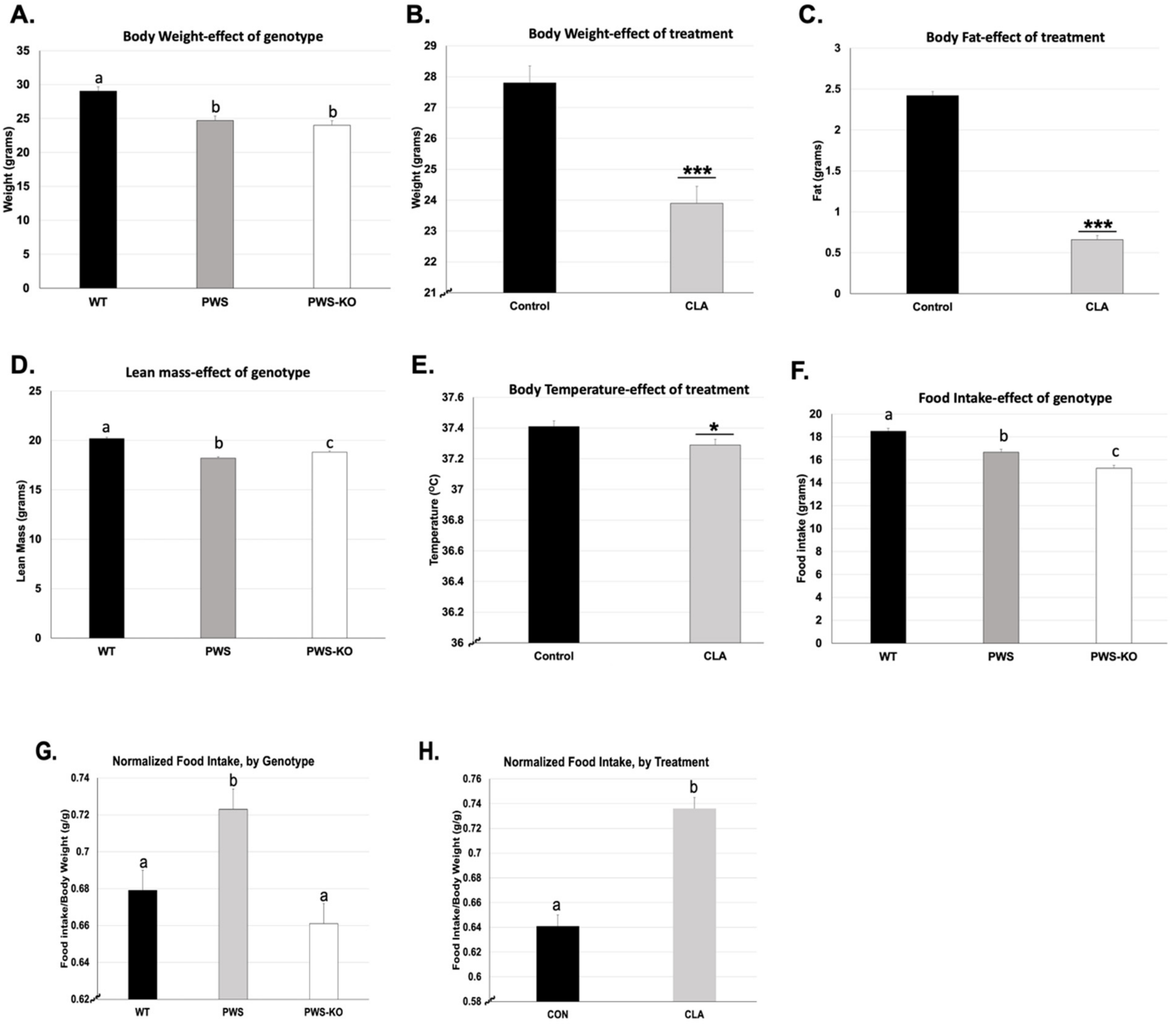

Figure S2: *Fasting Glucose with CLA diet-effect of treatment.* Glucose measurements were done at the end of the study (12 weeks). Post hoc analysis findings for fasting glucose revealed high significance (*** = *P <0.001*) for the effect of diet (genotypes groups by diet). Data are presented as mean +/- standard error of the mean. N=8 WT control, 8 WT CLA, 8 PWS control, 7 PWS CLA, 7 PWS-KO control, 8 PWS-KO CLA, grouped by diet.

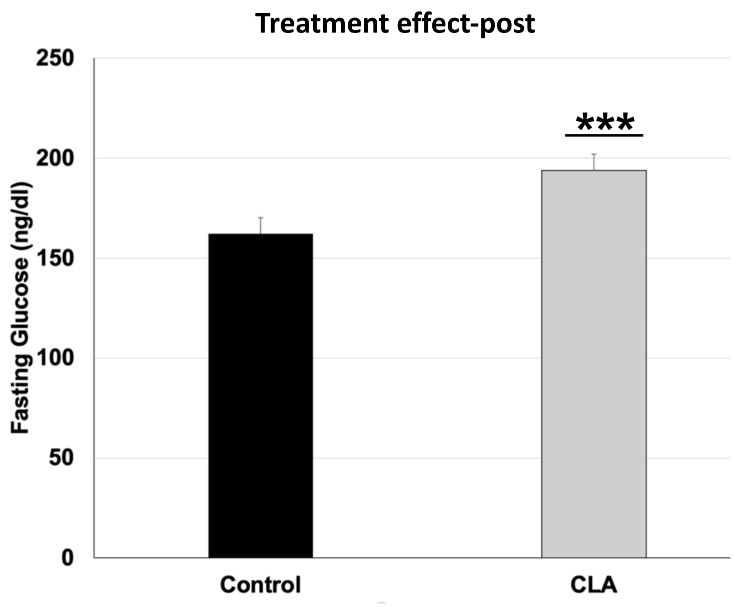

Figure S3: *Wheel Running (Post measures)* (A.) Post hoc analysis of genotype effects on wheel running were significant (*P=0.0008*) (B.) Treatment effects were non-significant (NS). N=8 WT control, N=8 WT CLA, N=7 PWS control, N=6 PWS CLA, N=8 PWS-KO control, N=7 PWS-KO CLA. ~~ used to indicate y-axis that are not at 0.

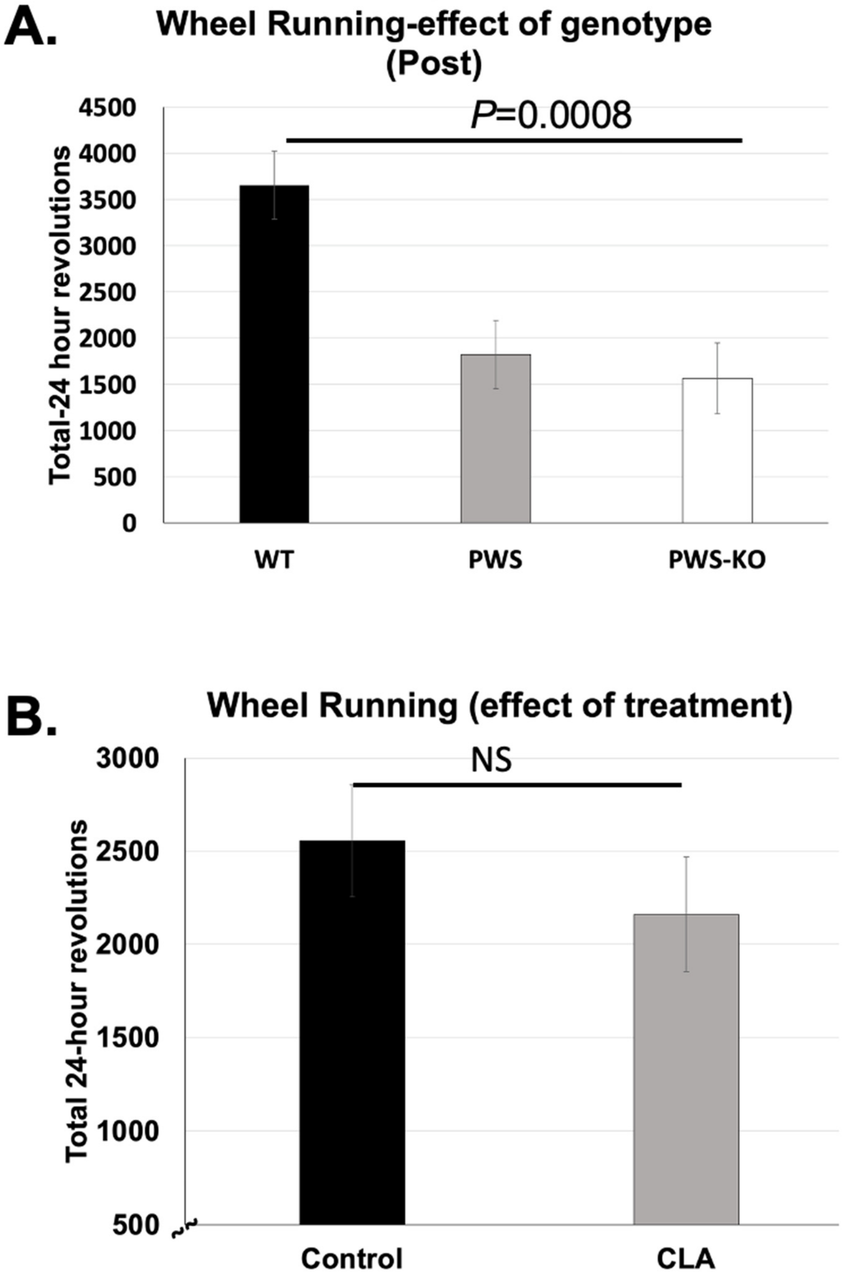

Figure S4: *Alpha Diversity in Microbiome Analysis.* Genotype and treated effects, and interactions are shown for each analysis. * = *P* <0.05, for diet interactions. The actual *P*-values are shown below each figure.

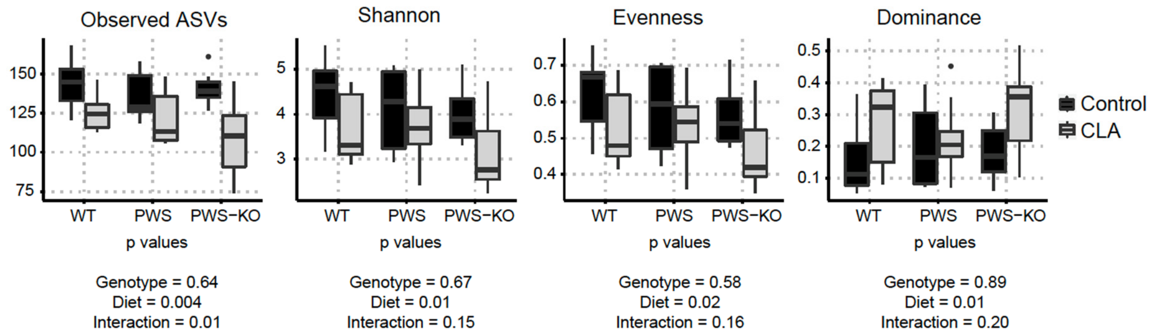

Supplement: Supplementary file 1 [file nutrients-14-00860-s001.zip › Supplemental Figures.pdf]
