# Supplementary material for: Dietary Conjugated Linoleic Acid Reduces Body Weight and Fat in Snord116m+/p− and Snord116m−/p− Mouse Models of Prader–Willi Syndrome
Source: Nutrients. 2022 Feb 18;14(4):860. doi: 10.3390/nu14040860 (PMC8880678; doi:10.3390/nu14040860)
Supplement: Supplementary file 1 [file nutrients-14-00860-s001.zip › Supplementary Data Files.pdf]

Supplementary Data Files

Supplementary Data Files provide whole transcript/gene data from the RNA-seq study, presented as individual Excel files online.

Supplementary Data Table S1a: List of differentially regulated genes in Excel format. Each comparison (genotype and treatment) is listed on a different sheet, and genes are organized in increasing *P*-value order. Each comparison of condition or genotype was analyzed using three different methods: DeSeq2, EdgeR, and Limma-voom. **A.** Control versus CLA (treatment analysis, all genotypes); **B.** WT control versus WT CLA treatment; **C.** PWS control versus PWS CL -treatment; **D.** WT versus PWS (genotype analysis, all treatments); **E.** WT control versus PWS control; **F.** WT CLA treatment versus PWS CLA treatment.

Supplementary Data Table S2: DeSeq2 results quantified to transcript levels. **A.** Control versus CLA (treatment analysis, all genotypes); **B.** WT control versus WT CLA treatment; **C.** PWS control versus PWS CL -treatment; **D.** WT versus PWS (genotype analysis, all treatments); **E.** WT control versus PWS control; **F.** WT CLA treatment versus PWS CLA treatment.

Supplementary Data Table S3: DeSeq2 results quantified to gene levels. **A.** Control versus CLA (treatment analysis, all genotypes); **B.** WT control versus WT CLA treatment; **C.** PWS control versus PWS CL -treatment; **D.** WT versus PWS (genotype analysis, all treatments); **E.** WT control versus PWS control; **F.** WT CLA treatment versus PWS CLA treatment.
