# Supplementary material for: Dietary Conjugated Linoleic Acid Reduces Body Weight and Fat in Snord116m+/p− and Snord116m−/p− Mouse Models of Prader–Willi Syndrome
Source: Nutrients. 2022 Feb 18;14(4):860. doi: 10.3390/nu14040860 (PMC8880678; doi:10.3390/nu14040860)
Supplement: Supplementary file 1 [file nutrients-14-00860-s001.zip › Supplementary Tables.pdf]

Supplementary Tables

Supplementary Tables provide GO-pathway analysis from the RNA seq study and are presented as standard tables.

Supplementary Table S1: Molecular pathway analysis; CLA vs Control (all genotypes). Analyzed via MouseMine: http://www.mousemine.org/mousemine/begin.do.

**Gene Ontology Enrichment-Biological Process**	**Adjusted p-Value** (Holm-Bonferroni)
Cellular metabolic process [GO:0044237]	0.001532
rRNA processing [GO:0006364]	0.008305
Organophosphate metabolic process [GO:0019637]	0.008949
rRNA metabolic process [GO:0016072]	0.015735
Cellular component organization or biogenesis [GO:0071840]	0.020753
**Gene Ontology Enrichment-Anatomy**	**Adjusted p-Value** (Holm-Bonferroni)
Telencephalon	0.016790
Retina	0.038596
Eye posterior segment	0.039956
Peripheral nervous system	0.042822
**Gene Ontology Enrichment-Mammalian Phenotype**	**Adjusted p-Value** (Holm-Bonferroni)
Mortality/aging [MP:0010768]	6.55E-06
Preweaning lethality [MP:0010770]	1.40E-05
Abnormal survival [MP:0010769]	2.12E-05
Embryonic lethality prior to tooth bud stage [MP:0013293]	6.05E-05

Prenatal lethality [MP:0002080]	1.84E-04
Embryonic lethality [MP:0008762]	1.85E-04
Embryonic lethality prior to organogenesis [MP:0013292]	2.56E-04
Abnormal behavior [MP:0004924]	0.029506
Behavior/neurological phenotype [MP:0005386]	0.029506

Supplementary Table S2: Molecular pathway analysis; CLA vs Control (WT genotype only). Analyzed via MouseMine: http://www.mousemine.org/mousemine/begin.do.

**Gene Ontology/Pathway**	**Adjusted p-Value** (Holm-Bonferroni)
Cellular response to starvation [GO:0009267]	0.007261
Cellular responses to stress	0.01229
Cellular responses to stimuli	0.01295
Response to starvation [GO:0042594]	0.015332
Cellular response to nutrient levels [GO:0031669]	0.020484

Supplementary Table S3: Molecular pathway analysis; PWS (*Snord116*$^{m+/p-}$) versus WT (both diets). Analyzed via MouseMine: http://www.mousemine.org/mousemine/begin.do.

**Gene Ontology/Pathway**	**Adjusted p-Value** (Holm-Bonferroni)
RBM3/CIRBP, RNA recognition [IPR034278]	0.020798
Abnormal bone mineral content [MP:0010122]	0.046985
